# Alterations of bacterial communities of vocal cord mucous membrane increases the risk for glottic laryngeal squamous cell carcinoma

**DOI:** 10.7150/jca.54221

**Published:** 2021-05-13

**Authors:** Zhen Dong, Chunming Zhang, Qinli Zhao, Hui Huangfu, Xuting Xue, Shuxin Wen, Yongyan Wu, Wei Gao, Binquan Wang

**Affiliations:** 1 Shanxi Key Laboratory of Otorhinolaryngology Head and Neck Cancer, First Hospital of Shanxi Medical University, Taiyuan 030001, China.; 2 Department of Otolaryngology Head & Neck Surgery, First Hospital of Shanxi Medical University, Taiyuan 030001, China.; 3 Shanxi Province Clinical Medical Research Center for Precision Medicine of Head and Neck Cancer, First Hospital of Shanxi Medical University, Taiyuan 030001, China.; 4 Department of Otolaryngology Head & Neck Surgery, Shanxi Bethune Hospital, Taiyuan 030032, China.; 5 Key Laboratory of Cellular Physiology, Ministry of Education, Shanxi Medical University, Taiyuan, 030001, China.; 6 Department of Biochemistry & Molecular Biology, Shanxi Medical University, Taiyuan 030001, China.; 7 Department of Cell biology and Genetics, Basic Medical School, Shanxi Medical University, Taiyuan 030001, China.

**Keywords:** microbiota, bacterial communities, vocal cord, glottic laryngeal squamous cell carcinoma, 16S rRNA sequences.

## Abstract

Bacteria are among the important factors that play a role in the balance of human health, and their relationship with some tumors has been well established. However, the association between bacteria colonizing the vocal cords and glottic laryngeal squamous cell carcinoma (GLSCC) remains unclear. Here, we investigated whether bacterial communities of the vocal cord mucous membrane play a role in the development of GLSCC. We collected tumor tissue and normal adjacent tissue (NAT) samples from 19 GLSCC patients, and the bacterial communities were compared with control samples (control) from 21 vocal cord polyps using 16S rRNA high-throughput pyrosequencing. We detected 41 phyla, 93 classes, 188 orders, 373 families, and 829 genera in the vocal cord mucous membrane. A comparison of the bacterial communities in the NAT samples showed higher α‐diversity than in the tumor samples. In the tumor samples, seven groups of bacteria, i.e., the phylum Fusobacteria, the class Fusobacteriia, the order Fusobacteriales, the family Fusobacteriaceae, and the genera *Fusobacterium*, *Alloprevotella*, and *Prevotella*, were significantly enriched, as revealed by linear discriminant analysis coupled with effect size measurements (LEfSe). However, bacteria from the phylum Firmicutes were most significantly enriched in the vocal cord polyp tissues. These findings suggest alterations in the bacterial community structure of the vocal cord mucous membrane of GLSCC patients and that seven groups of bacteria are related to GLSCC, indicating that imbalances in bacterial communities increase the risk for the development of GLSCC.

## Introduction

The human body is a carrier of microorganisms. These microorganisms are mainly distributed in the digestive tract, the respiratory tract, the urinary tract, and on the skin; there are about 10^14^ bacterial cells in each of these organ systems, which is about 10 times higher than the number of somatic cells in adults 1. These bacteria can exchange energy and materials and transmit information with the host. As such, they play a major role in human health by providing nutrition, immunity, growth stimulation, and biological antagonism to the host 2. Although the microbiomes living in symbiosis with the human body have long been regarded as key to elucidating various human disorders, we are only beginning to comprehend their equally important function in the maintenance of human health. As early as 2008, the National Institutes of Health isolated microbial DNA samples from 18 different body regions of more than 200 healthy subjects and performed 16S rRNA tag sequencing and shotgun metagenomic sequencing to study “the similarities and differences between individuals and body sites, and through time the numbers and types of microbes and what role they play in human health” 3. This project, named the Human Microbiome Project (HMP), has served as a solid foundation for investigating the human microbiome.

In 1984, it was found that *Helicobacter pylori* is a causative pathogen of gastric tumors 4. Thus, a new research direction, namely, the relationship between microorganisms and tumor development, was established. In recent years, increasing evidence has supported the relationship between specific microorganisms and systemic tumors, including reproductive, intestinal, and oral tumors 5-8. Allavena confirmed that 15-20% of malignant tumors are associated with microbial infections 9. These studies suggest that there may also be a close relationship between bacterial communities and human tumors.

Laryngeal squamous cell carcinoma (LSCC) is a common head and neck malignant tumor and accounts for 25% of head and neck cancers and 2-3% of the total number of cancers 10. Experts have explored the pathogenic factors of LSCC. Their studies usually focus on tobacco use, gastroesophageal reflux, radiation exposure, diet type, occupation, and genetic inheritance 11-13. But could there be a relationship between LSCC and bacteria that are closely related to human diseases? Gong et al 14-15 studied the correlation between the microbial structure of laryngeal mucosa and LSCC through high-throughput pyrosequencing. Their results revealed that the characteristics of laryngeal microflora in patients with laryngeal cancer had changed compared with normal individuals. Glottic LSCC (GLSCC) is a malignant tumor originating from the vocal cord, accounting for about 60% of LSCC cases 16. The composition and abundance of the bacterial communities of the human vocal cord mucous membrane and the possible correlation between the bacterial community and GLSCC remain unclear. To obtain insights into this relationship, we selected 19 patients with GLSCC as well as 21 patients with vocal cord polyps as controls. Through high-throughput sequencing analysis of variable region 3 (V3) and variable region 4 (V4) of 16S rRNA, we characterized the bacterial communities on the vocal cord of patients diagnosed with GLSCC and on vocal polyps of control patients and compared the bacterial community structures between tumor tissue samples, normal adjacent tissue (NAT) samples, and vocal cord polyps (control). The present study aimed to obtain a better understanding of the ecological conditions of bacterial communities in the vocal cord mucous membrane of GLSCC patients and control individuals and to determine whether these conditions play an important role in the development of GLSCC.

## Materials and methods

### Subject recruitment and selection

We collected samples from 40 surgical patients at the Department of Otolaryngology and Head and Neck Surgery of the First Hospital of Shanxi Medical University. The study subjects included 19 patients with GLSCC and 21 patients with vocal cord polyps; these individuals were recruited between June 2018 and November 2019. We enrolled 7 females and 33 males, with ages ranging from 30 to 78 years (average: 57.4±14.2 years). Smoking and drinking were also recorded for each subject. Tumor stage was assessed based on the International Union Against Cancer TNM classification system, 6th Edition 17. Patients who were treated with any antibiotics, immunosuppressors, hormones, and/or antimycotics within the past 30 days were excluded from the study. The GLSCC patients underwent partial or total laryngectomy, whereas the control subjects with vocal cord polyps underwent suspension laryngoscope surgery. The size of GLSCC tissue, NAT, and vocal cord polyp (control) tissue samples was 3 mm × 3 mm × 1 mm. The study protocol was approved by the Committee on Ethics of the First Hospital of Shanxi Medical University, and informed consent was signed by all participants.

### Sample collection

Tumor tissues were collected from the surface of each tumor site from subjects with GLSCC, whereas NATs were obtained from an area at least 1 cm from the site of the tumor 18. The control tissues were collected from the polyp superficial layer of patients diagnosed with vocal cord polyps; this is the only ethical method of collecting non-tumor tissues from the vocal cord of non-tumorous subjects and has been described in an earlier study 19. Although vocal cord polyps are not considered healthy tissues, earlier studies have indicated that they may be utilized as meaningful control samples 14, 15, 20, 21. The study performed by Hanshew et al. showed that tissue and non-invasive swab sampling methods exhibited similar performance 22. Notably, a majority of previous studies adopted the tissue sampling method for high-throughput pyrosequencing. A total of 19 tumor tissues and 18 NAT samples were collected. One NAT sample was excluded because the edge of the tumor tissue was less than 1 cm. To avoid contamination, all samples were obtained in a laminar flow operating room immediately after the operation. Postoperative histopathological examination confirmed the diagnosis of each patient. All samples were collected in microcentrifuge tubes (Axygen, Shanghai) and stored at -80°C until DNA extraction.

### DNA extraction and PCR amplification

We used the QIAamp DNA Mini Kit (QIAGEN, Germany) to extract total genomic DNA, following the manufacturer's instructions. DNA quality or quantity was determined by NanoDrop and agarose gels. The extracted DNA was diluted to a concentration of 1 ng/μL and then stored at -20°C. With the use of diluted DNA as a template and Ex Taq PCR mixture (Takara, Dalian), PCR was carried out to amplify the V3-V4 region of the bacterial 16S rRNA gene with two specific bacterial primers: 343F (5′-TACGGRAGGCAGCAG-3′) and 798R (5′-AGGGTATCTAATCCT-3′) 23. Amplicon quality was assessed using gel electrophoresis, purified using Agencourt AMPure XP beads (Beckman Coulter, CA, U.S.A), and then subjected to another round of PCR amplification. After another round of purification with AMPure XP beads, we quantified the final amplicon with a Qubit dsDNA analysis kit (Thermo Fisher Scientific Inc., CA, U.S.A). Then, the purified PCR products were analyzed by sequencing.

### Sequencing and data analyses

Sequencing of the 16S rRNA genes was conducted at OEbiotech Co. Ltd. (Shanghai, China). The amplified 16S rRNA genes from various samples were mixed in equal proportions and then subjected to pyrophosphate sequencing using an Illumina MiSeq platform. The raw sequencing data were transformed into the FASTQ format. Then, trimmomatic software 24 was employed to pretreat the paired-end reads to identify and exclude any ambiguous bases. A sliding window trimming method was used to remove low-quality sequences with an average quality score of <20. After trimming, FLASH software was employed to assemble paired-end reads 25. The assembly conditions were as follows: minimum overlap of 10 bp, maximum overlap of 200 bp, and 20% maximum mismatch rate. Further denoising of the sequences was performed by excluding ambiguous, homologous, or <200-bp reads. Reads with 75% of the bases > Q20 were retained. Then, the chimera reads were examined and removed. These two steps were performed with QIIME software (ver. 1.8.0) 26. Vsearch software using a 97% similarity cutoff was employed for primer sequence removal as well as clustering of clean reads to obtain operational taxonomic units (OTUs) 27. The QIIME software package was used to select representative reads for each OTU. A Ribosomal Database Project (RDP) classifier was employed to annotate all representative reads and deliver them to the Silva database (ver. 123) with a confidence of 70% 28.

Richness was assessed based on the Chao1 value. Shannon and Simpson diversities were evaluated with the non-parametric Shannon and Simpson indices. The Chao1 value, Good's coverage index, the Simpson index, and the Shannon index were calculated using the mothur program 29. Based on the RDP 30, sequences were assigned to phylogenetic classifications using the online RDP classifier. These sequences were assigned to the staging unit with a bootstrap cutoff of 80%. The heatmap.2 software package was used to generate a heatmap that shows the distance of the sample clusters, and UniFrac software was used to analyze the richest sequences at the phylum, class, family, order, and genus levels 31. The data are presented as the median and mean. Analysis was performed using SPSS23.0 (SPSS Inc., Chicago, IL, USA). The Kruskal-Wallis test was employed to compare groups. To visualize the separation of objects using pairwise distances, a principal coordinate analysis (PCoA) graph was constructed to show the first two principal coordinates 32. To analyze the differences in the microbial composition of vocal cords among the three groups, we used the permutational multivariate analysis of variance (PERMANOVA) “Adonis” function of weighted UniFrac distance metrics. *P*< 0.05 was considered to indicate statistical significance. Linear discriminant analysis (LDA) coupled with effect size measurements (LEfSe) was performed to identify biomarkers among groups 33.

## Results

### Clinical characteristics of subjects and sequencing data quality

In this study, 19 patients with GLSCC (18 males, 1 female) and 21 patients with vocal cord polyps (15 males, 6 females) were included. Their clinical information, including age, tumor size and classification, and lymph node metastasis, is presented in Table 1.

We obtained 1,363,289 valid tags after processing the bacterial V3-V4 sequencing data of the 16S rRNA genes. The average number of valid tags of each sample was 23,514 ± 10,162 (ranging from 7,866 to 51,154), and the average length was 427 ± 7 bp (ranging from 398 to 436 bp). After 97% pairwise identical cut-off sequences were assigned to species OTUs, we obtained 10,554 OTUs and 3 core OTUs (Figure S1-S2). To provide the features of sequence reads, the coverage percentage (Good's coverage), richness estimation (Chao1), and diversity indices (Shannon and Simpson) based on the estimated OTUs were used (Table 2).

### Bacterial community profiles in vocal cord mucosa

**Figure 1:**
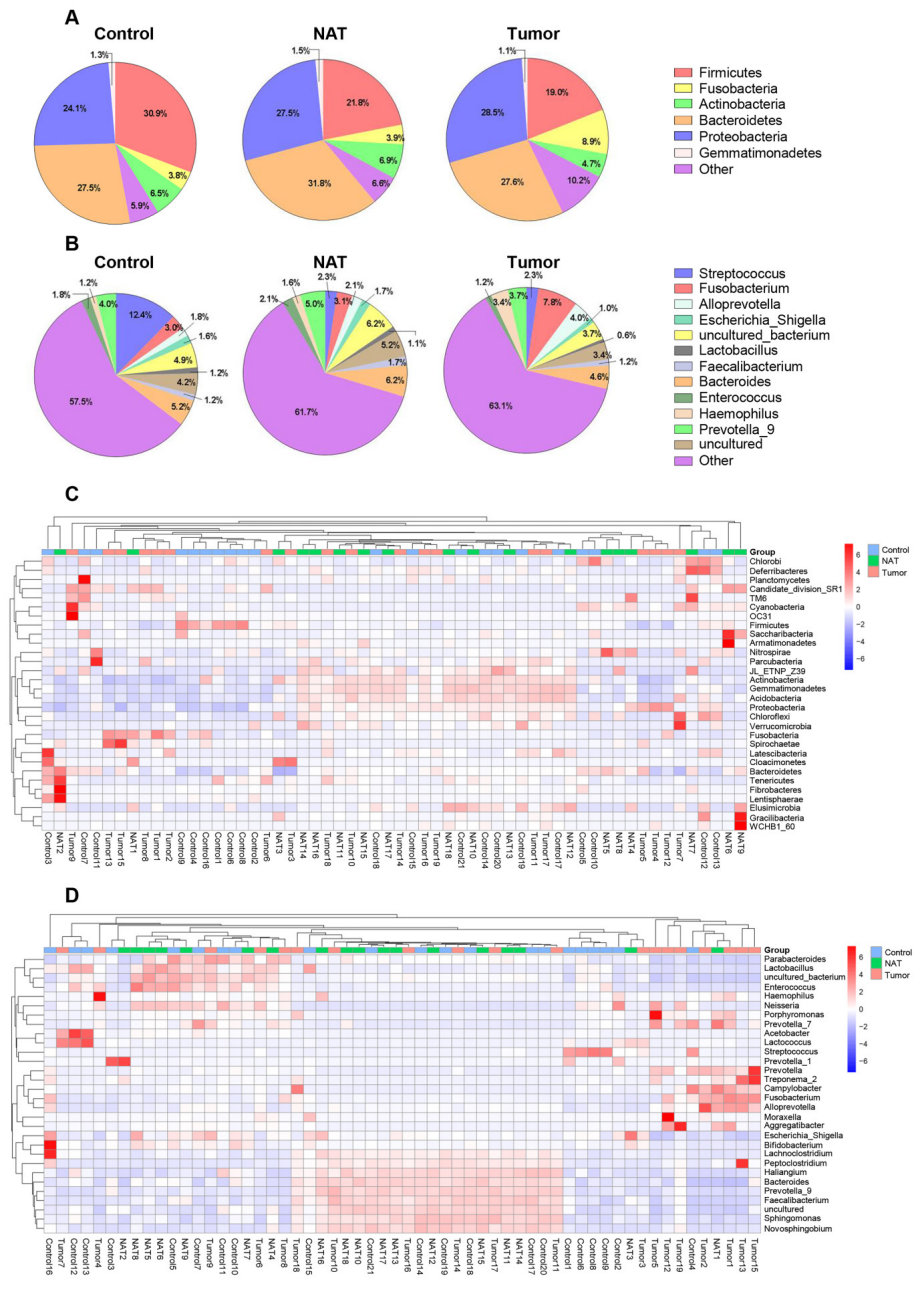
Relative abundance of major bacterial phyla (A) and genera (B) whose proportions were greater than 1% in the vocal cords. The values are the mean sequence abundances in various groups and levels. Heatmap showing the relative densities of 30 of the most abundant phyla (C) and genera (D) of vocal cord tissue samples. Hierarchical dendrogram depicting the taxonomic assignments of vocal cord samples. The cluster branch groups above represent samples from different groups. The cluster tree on the left represents the cluster of genera. The legend in the upper-right corner of the figure indicates the colors that represent the relative abundances of genera in every sample (presented as a percentage of the total 16S rRNA sequences). Orange indicates a higher relative abundance of the genera, and blue shows a lower relative abundance. The tumor tissue and normal adjacent tissue (NAT) samples were taken from GLSCC patients, and the control tissue was sampled from subjects with vocal cord polyps.

In total, we found 41 phyla, 93 classes, 188 orders, 373 families, and 829 genera in the vocal cords of the study subjects. The predominant phyla were Firmicutes (mean: 24.2%, range: 8.2-60.1%), Fusobacteria (mean: 5.5%, range: 0.01-28.4%), Bacteroidetes (mean: 28.9%, range: 10.5-55.3%), Proteobacteria (mean: 26.6%, range: 9.4-53.6%), and Actinobacteria (mean: 6.1%, range: 0.5-14.3%). The most prevalent communities of genera were the following: *Streptococcus* (mean: 5.9%, range: 0.2-39.0%), *Fusobacterium* (mean: 4.6%, range: 0.1-14.1%), *Prevotella_9* (mean: 4.2%, range: 0.2-9.9%), *Bacteroides* (mean: 5.3%, range: 0.4-8.8%), *Alloprevotella* (mean: 2.6%, range: 0-15.4%), *Haemophilus* (mean: 2.0%, range: 0-11.2%), and *Enterococcus* (mean: 1.7%, range: 0.1-8.8%). The relative abundance of various phyla and genera in each sample is shown in Figure 1. The predominant phyla in the respective control tissue, NAT, and tumor tissue were Firmicutes (30.9%, 21.8%, and 19%), followed by Fusobacteria (3.7%, 3.9%, and 8.9%), Bacteroidetes (27.5%, 31.8%, and 26.4%), Proteobacteria (24.1%, 27.5%, and 28.5%), and Actinobacteria (6.5%, 6.9%, and 4.7%) (Figure 1A). The relative abundances of the major bacterial genera in the vocal cord mucosa were *Streptococcus* (12.3%, 2.3%, and 2.3%), *Fusobacterium* (2.9%, 3.1%, and 7.8%), *Prevotella_9*(4.0%, 5.0%, and 3.7%), *Neisseria* (1.7%, 1.3%, and 1.4%), and *Alloprevotella* (1.8%, 2.0%, and 3.9%) (Figure 1B). Based on relative abundance, a colored heatmap was constructed to visualize the 30 most common phyla and genera in the three groups (tumor tissue, NAT, and control tissue) (Figure 1C and D).

### Comparison of bacterial community diversity in GLSCC patients and control patients

**Figure 2:**
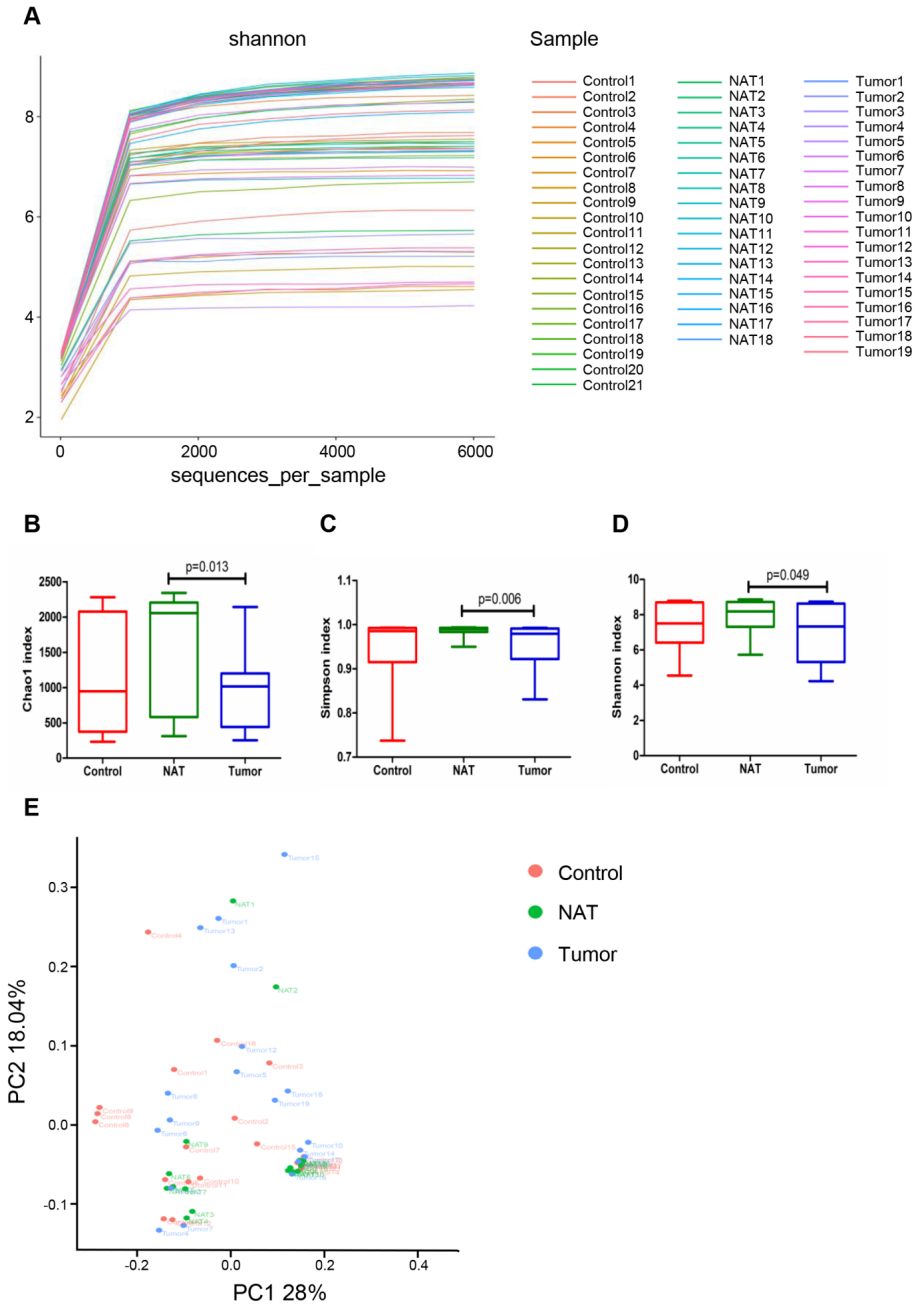
Comparative analyses of the vocal cord bacterial communities of the three groups of tissue samples. Alpha rarefaction plot (A), Chao1 (B), Simpson index (C), and Shannon index (D) of the samples from the tumor tissue, the normal adjacent tissue (NAT), and control tissue were compared. These comparisons were not marked because *P*> 0.05 between groups. (E) Principal coordinate analysis (PCoA) using the weighted UniFrac distance of the vocal cord microbiota among the study participants. The weighted UniFrac distance significantly varied among the three groups (weighted UniFrac R^2^ = 0.067, *P*< 0.05).

Rarefaction analysis (Figure 2A) indicated that the OTUs were almost saturated to a platform with a genetic distance of 2-3% for these 58 samples, indicating that most of the bacterial composition was included and our sequencing depth was sufficient. We detected higher bacterial richness in the NATs than in the tumor samples (Chao1, *P*< 0.05) (Figure 2B). The top 15 contents affiliated to bacteria identified in the NAT samples were listed in table S1. Additionally, we obtained higher indices for Simpson and Shannon diversities in the NAT samples relative to the tumor samples (Simpson, *P*< 0.05 and Shannon, *P*< 0.05) (Figure 2C and D). However, no significant differences in α‐diversity indices were observed between the tumor and control samples (Figure 2B-D). To estimate the overall differences among the microbial communities of the three groups with respect to β-diversity, we used weighted UniFrac to evaluate dissimilarities, which were further visualized in the PCoA diagram. The first two axes (PC1 and PC2) accounted for 28% and 18.04%, respectively, of the observed total variation in the vocal cord bacterial communities. Based on distance matrices (Adonis), PERMANOVA showed that inter-group bacteria had a marked contribution (weighted UniFrac R^2^ = 0.067, *P*< 0.05). These findings showed that the spatial structure of the bacterial communities in the tumor samples varied significantly from that in the control samples based on the PCoA results (Figure 2E).

### Changes in the vocal cord bacterial community structure at the phylum level

**Figure 3:**
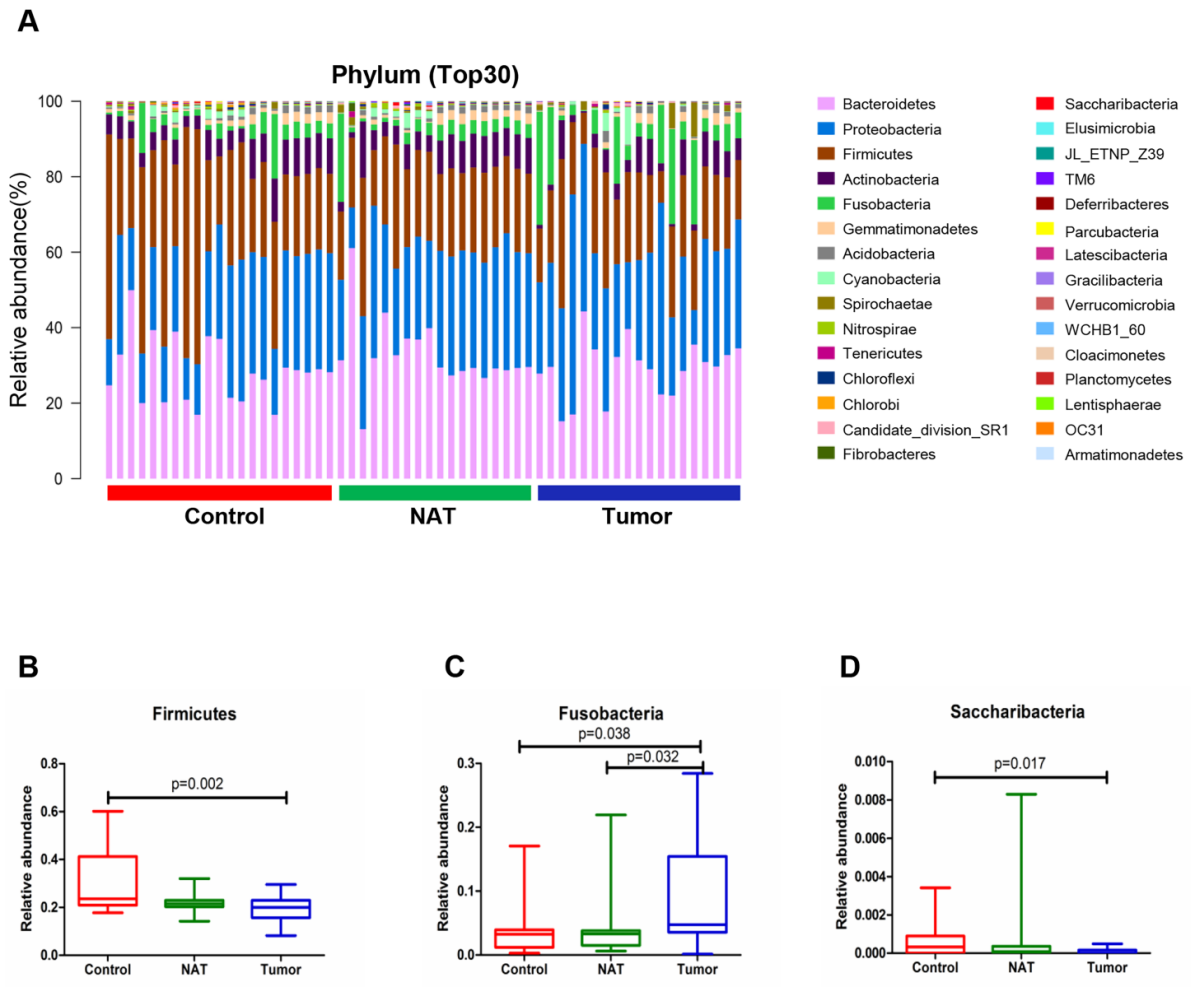
Alterations in the vocal cord bacterial community compositions at the phylum level. (A) Histogram of the bacterial community structure at the phylum level (top 30). Each column represents a vocal cord sample, and each color represents an individual phylum. (B) Firmicutes. (C) Fusobacteria. (D) Saccharibacteria. Comparisons among groups were performed using Kruskal-Wallis tests. These comparisons were not marked because *P*> 0.05 between groups.

The relative abundance graph representing the vocal cord bacterial community composition at the phylum level is presented in Figure 3A. An increase in the population size for the phylum Fusobacteria was observed in the tumor samples relative to the NATs and the control samples (*P*< 0.05) (Figure 3C). However, the phyla Firmicutes and Saccharibacteria showed a decrease in population size in the tumor sample compared with the control sample (*P*< 0.05) (Figure 3B and D).

### Changes in the vocal cord bacterial community structure at the class level

**Figure 4:**
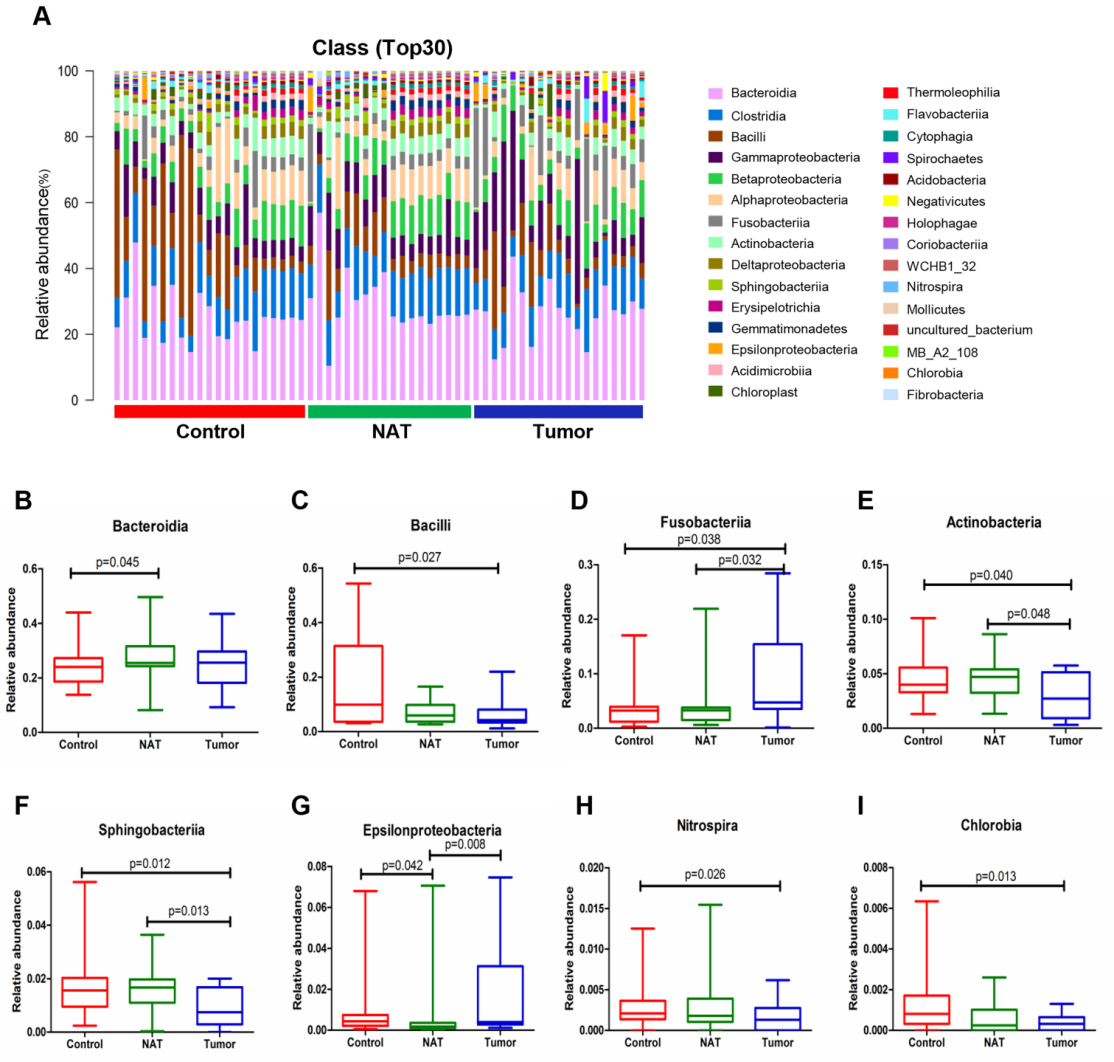
Alterations in the vocal cord microbiota composition. (A) Histogram of the bacterial community structure distribution at the class level (top 30). Each column represents a vocal cord sample, and each color represents an individual class. (B) Bacteroidia. (C) Bacilli. (D) Fusobacteriia. (E) Actinobacteria. (F) Sphingobacteria. (G) Epsilonproteobacteria. (H) Nitrospira. (I) Chlorobia. Comparisons among groups were performed using Kruskal-Wallis tests. These comparisons were not marked because *P*> 0.05 between groups.

The relative abundance graph of the vocal cord bacterial community composition at the class level is shown in Figure 4A. The results show that the level of Fusobacteriia was significantly higher in the tumor sample relative to the control and NAT samples (Figure 4D). In addition, we found that the tumor samples had significantly higher levels of Bacilli, Actinobacteria, Sphingobacteriia, Nitrospira, and Chlorobia compared with the control samples (Figure 4C, E, F, H, and I). Furthermore, the levels of Fusobacteriia, Actinobacteria, Sphingobacteriia, and Epsilonproteobacteria were significantly altered in the tumor samples compared with the NAT samples (Figure 4D-G).

### Changes in the vocal cord bacterial community structure at the order level

**Figure 5:**
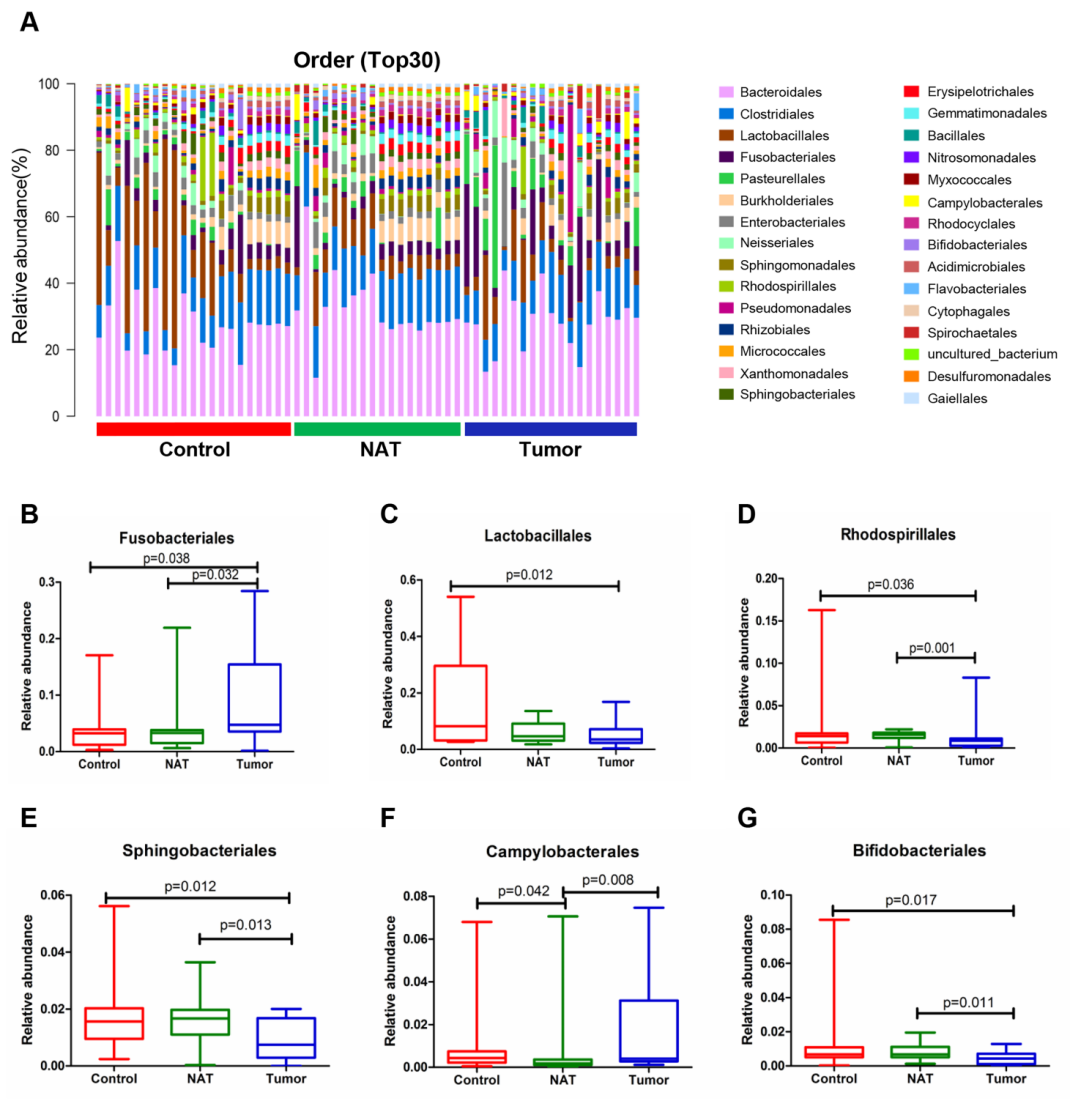
Alterations in the vocal cord microbiota composition at the order level. (A) Histogram of the bacterial community structure distribution at the order level (top 30). Each column represents a vocal cord sample, and each color represents an individual order. (B) Fusobacteriales. (C) Lactobacillales. (D) Rhodospirillales. (E) Sphingobacteriales. (F) Campylobacterales. (G) Bifidobacteriales. Comparisons among groups were performed using Kruskal-Wallis tests. These comparisons were not marked because *P*> 0.05 between groups.

The relative abundance graph of the vocal cord bacterial community composition at the order level is shown in Figure 5A. The relative abundances of Lactobacillales, Rhodospirillales, Sphingobacteriales, Campylobacterales, and Bifidobacteriales were significantly altered among the three groups. Further analysis showed that the relative abundances of Lactobacillales, Rhodospirillales, Sphingobacteriales, and Bifidobacteriales were markedly decreased in the tumor tissues compared to the control samples (Figure 5C-E and G). However, the level of Fusobacteriales in the tumor samples was higher than that in the control samples (Figure 5B).

### Changes in the vocal cord bacterial community structure at the family level

**Figure 6:**
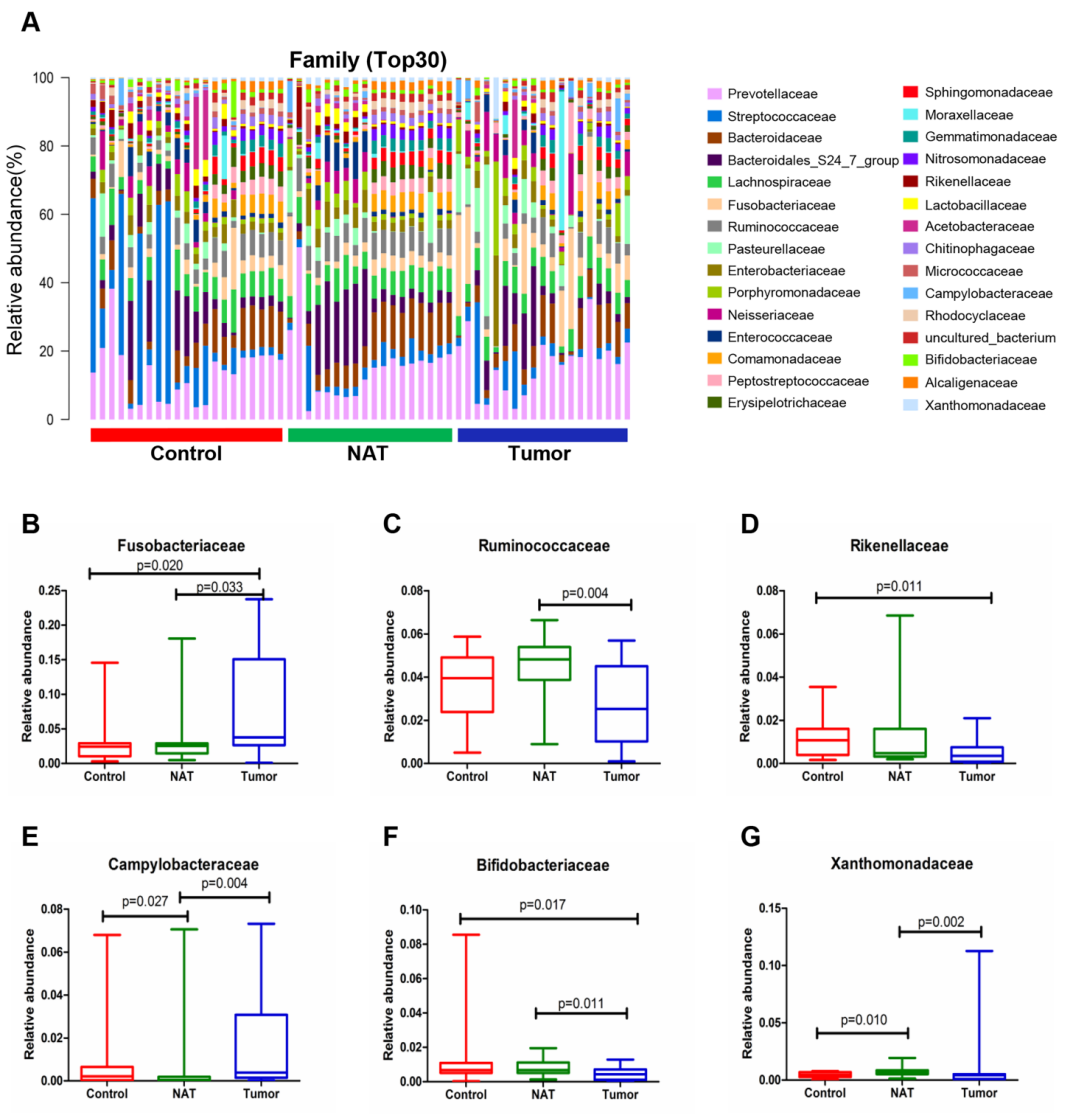
Alterations in the vocal cord microbiota composition at the family level. (A) Histogram of the bacterial community structure distribution at the family level (top 30). Each column represents a vocal cord sample, and each color represents an individual family. (B) Fusobacteriaceae. (C) Ruminococcaceae. (D) Rikenellaceae. (E) Campylobacteraceae. (F) Bifidobacteriaceae. (G) Xanthomonadaceae. Comparisons among groups were performed using Kruskal-Wallis tests. These comparisons were not marked because *P*> 0.05 between groups.

The relative abundance graph of the vocal cord bacterial community composition at the family level is shown in Figure 6A. The relative abundances of the families Fusobacteriaceae, Ruminococcaceae, Campylobacteraceae, Bifidobacteriaceae, and Xanthomonadaceae showed significant changes between the tumor samples and the NAT samples (*P*< 0.05) (Figure 6B, C, and E-G). The relative abundance of the family Fusobacteriaceae in the tumor samples was significantly higher than in the control samples (P < 0.05) (Figure 6B). However, the tumor tissues and NAT samples had significantly lower relative abundance of the families Rikenellaceae and Bifidobacteriaceae relative to the control samples (*P*< 0.05) (Figure 6D and F).

### Changes in the vocal cord bacterial community structure at the genus level

**Figure 7:**
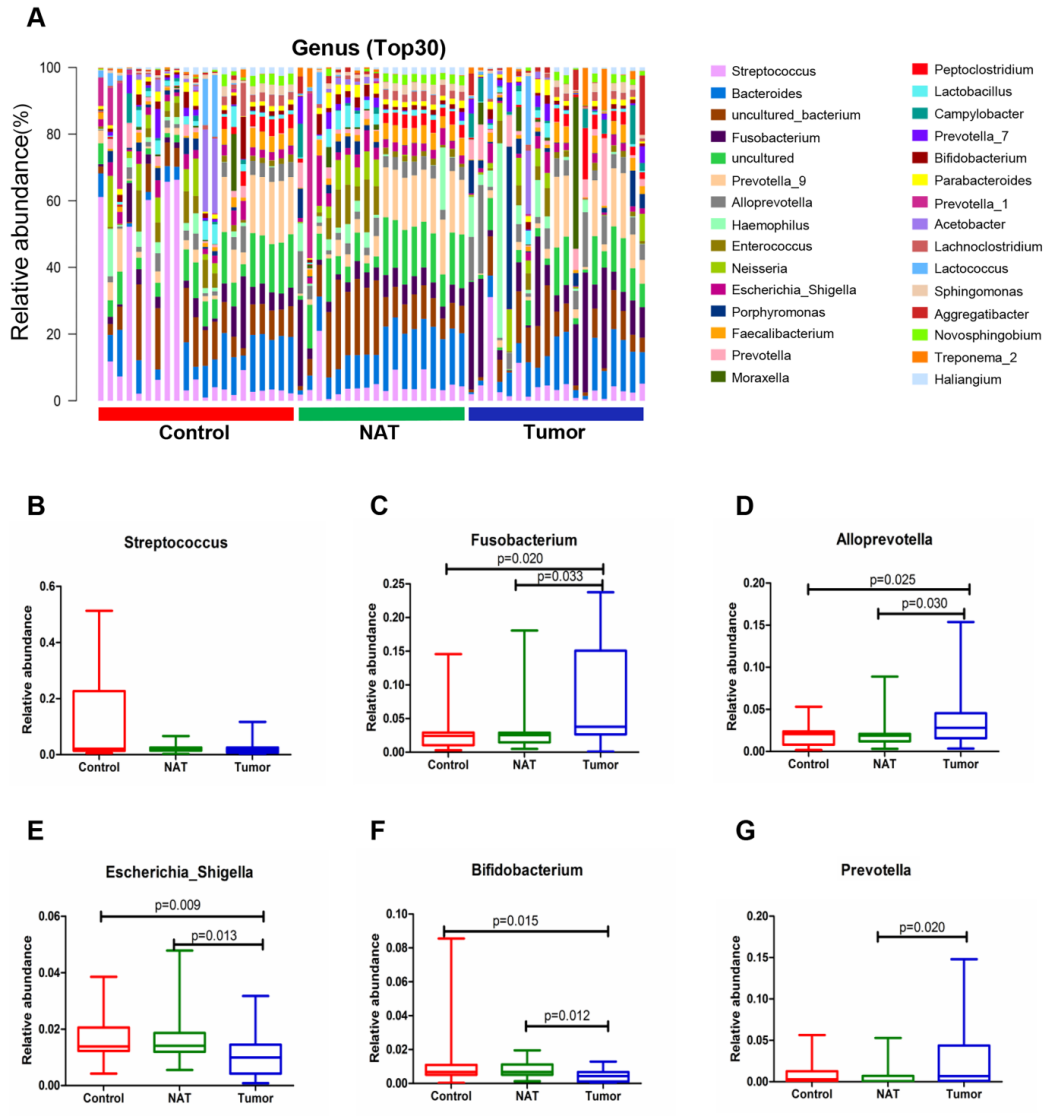
Alterations in the vocal cord microbiota composition at the genus level. (A) Histogram of the bacterial community structure distribution at the genus level (top 30). Each column represents a vocal cord sample, and each color represents an individual genus. (B) *Streptococcus*. (C) *Fusobacterium*. (D) *Alloprevotella*. (E) *Escherichia_Shigella*. (F) *Bifidobacterium*. (G) *Prevotella*. Comparisons among groups were performed using Kruskal-Wallis tests. These comparisons were not marked because *P*> 0.05 between groups.

The relative abundance graph of the vocal cord bacterial community composition at the genus level is shown in Figure 7A. The level of *Streptococcus* did not change significantly among the three groups (*P*> 0.05) (Figure 7B). Compared with the control samples, the relative abundances of the genera *Fusobacterium* and *Alloprevotella* were significantly increased in the tumor samples (*P*< 0.05) (Figure 7C and D). However, the relative abundances of the genera *Escherichia_Shigella* and *Bifidobacterium* were lower in the tumor tissues and NAT samples relative to the control samples (*P*< 0.05) (Figure 7E and F).

### Taxonomy-based comparisons of bacterial community groups

**Figure 8:**
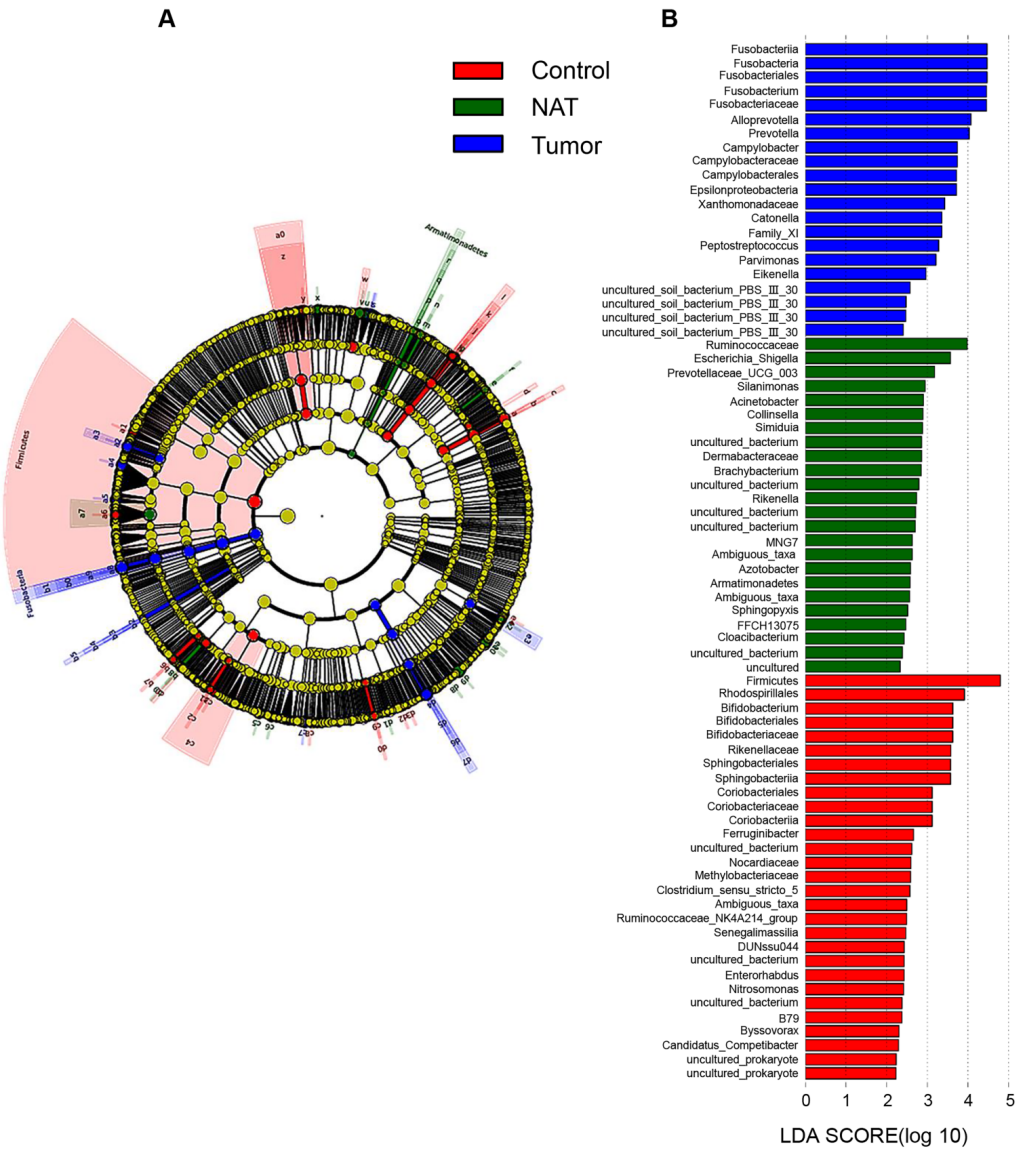
Linear discriminant analysis (LDA) coupled with effect size measurements (LEfSe) analysis. (A) Structure of the vocal cord microbiota. The cladogram depicts the relationships among microbiota taxonomic units of the bacteria from the phylum level to the genus level. The colors red, green, and blue represent bacterial taxonomic units that were abundant in vocal cord polyp tissue (control), normal adjacent tissue (NAT), and tumor tissue (tumor). (B) Histogram of LDA scores of taxonomic units demonstrating the contribution of different taxonomic units to the difference.

In our study, each group is presented in cladograms, and histograms of LDA scores of 2 or more were generated by LEfSe (Figure 8). The dominant phyla, classes, orders, families, and genera of the vocal cord bacterial communities changed obviously. In the tumor tissues, the phylum Fusobacteria, the class Fusobacteriia, the order Fusobacteriales, the family Fusobacteriaceae, and the genera *Fusobacterium*, *Alloprevotella*, and *Prevotella* were significantly enriched (LDA scores ≥ 4). However, Firmicutes was most significantly enriched in the vocal cord polyp tissue.

## Discussion

This study assessed the vocal cord microbiome of 19 patients with GLSCC and 21 controls. The results showed that there were significant differences in the vocal cord bacterial communities among the tumor, NAT, and control samples. Relative to the control group, in addition to the observation of different vocal cord microbiome structure patterns in GLSCC patients, the relative abundance of certain OTUs was significantly different between GLSCC and control patients. These results show that alterations in the bacterial community structure of the vocal cord mucous membrane are related to GLSCC. A combination of multiple bacterial OTUs may produce the same disease outcome 34. Among these three groups, there was no significant difference with respect to the relative abundance of several predominant genera. It is likely that these bacteria do not play a key role in this environment. However, it is generally believed that low-abundance microorganisms may play a major role in the human niche. They may play a key role in the responses to environmental alterations, and they may have a profound impact on the formation of the microenvironment over time by serving as a sustainable resource for genomic innovation 35. The relationship between bacterial species and GLSCC was not explored in our study because high-throughput sequencing is not suitable to accurately identify bacterial species. Further study is needed for species-level analysis.

We found that microbial richness (evaluated by Chao1), Simpson diversity, and Shannon diversity were significantly higher in tumor samples than in NAT samples. However, there was no significant variation in α-diversity between the tumor and control groups, which is not completely consistent with previous laryngeal community analyses using 16S rRNA marker genes 14. In fact, many factors can cause discrepancies in the α-diversity of these microbial communities, including the host's physiological state, experimental methods, the sample type, and the bioinformatics approach 36.

We observed consistent alterations of vocal cord microbiota communities across different taxonomic levels in GLSCC patients and vocal cord polyp patients. At the phylum level, Firmicutes, Bacteroidetes, Proteobacteria, Actinobacteria, and Fusobacteria were most abundant in the three groups, which is in nearly perfect agreement with a previous study 15. Compared to the control group, the population size of the phylum Firmicutes within tumor samples was reduced, which showed that Firmicutes may be a protective factor against tumors. At the genus level, the relative abundances of *Fusobacterium* and *Alloprevotella* were higher in the vocal cords of GLSCC patients, while that of *Escherichia_Shigella* decreased. The phylum Fusobacteria, the class Fusobacteriia, the order Fusobacteriales, the family Fusobacteriaceae, and the genera *Fusobacterium*, *Alloprevotella*, and *Prevotella* were significantly enriched in the tumor tissue (LDA scores > 4). Thus, these groups may be related to GLSCC.

*Fusobacterium* is a genus of the phylum Fusobacteria. *Fusobacterium* species are proinflammatory pathogens that modulate the tumor immune microenvironment through CD11b^+^ cell expansion, thus inducing inflammation and promoting tumorigenesis 37. Previous studies have confirmed that patients with colorectal, oral, and stomach cancer are enriched in *Fusobacterium* 38-40. From the healthy controls to oral squamous cell carcinoma stages 1 to 4, the level of Fusobacteria significantly increased with oral cancer progression. At the genus level, the relative abundance of *Fusobacterium* increased, whereas the abundances of *Streptococcus*, *Haemophilus*, *Porphyromonas*, and *Actinomyces* decreased with cancer progression 41. *Fusobacterium nucleatum* (F.n) is crucial to the proliferation and invasion of colorectal tumor cells. Ohkusa et al reported that *Fusobacterium* can gain entry into cells and trigger proinflammatory cytokine secretion 42. Some studies using quantitative PCR (qPCR) have shown that the mRNA levels of interleukin-8 (IL-8) and IL-6 in colorectal cancer (CRC) cells that were penetrated by F.n were significantly increased 43,44. Elevated IL-8 and IL-6 mRNA levels enhance tumor cell proliferation. At the same time, qPCR and immunohistochemistry showed that the expression levels of nuclear phosphorylated NF-κB p65 were higher in clinical CRC samples than in control subjects 45,46. These findings are related to the results of Yang et al., who showed that F.n promotes the proliferation of CRC cells via activation of TLR4 signaling to NF-κB 47. According to Mima's study 6, F.n is expected to be used as a biomarker for CRC screening. Thus, F.n can influence the progression of CRC. However, additional molecular studies on the relationship between F.n and the development of GLSCC have not been conducted.

In 2013, the anaerobic genus *Alloprevotella* was first isolated from the human oral cavity by Julia Downes 48. *Alloprevotella* was also found to be more abundant in gastric adenocarcinoma samples than in matched control samples 49. It was recently reported that the periodontal pathogen *Alloprevotella* was enriched in oral cavity squamous cell tumors 50. We hypothesize that *Alloprevotella* migrates from the oral cavity to the vocal cords, affecting the progression of GLSCC.

*Streptococcus* is a Gram-positive genus belonging to phylum Firmicutes, which consists of more than 50 species. Different species of *Streptococcus* have been connected with pharyngitis, pneumonia and upper respiratory tract infections, otitis media, and sepsis. Several studies involving the normal esophageal microbiome have also shown the predominance of *Streptococcus* 51, 52. Similarly, its abundance is high in the healthy salivary microbiome in children and adults 53. Jetté compared healthy laryngeal microbiome samples with samples from benign vocal cord diseases by pyrosequencing of the 16S rRNA gene and revealed higher abundance of *Streptococcus* in benign vocal fold diseases 54. Gong et al. 14, 15 confirmed that *Streptococcus* is highly abundant in laryngeal carcinoma tissues, but our study found that there was no significant difference between GLSCC and vocal cord polyp samples with respect to the relative abundance of *Streptococcus*. This difference may be attributed to variations in experimental methods, research sites, and statistical methods.

The microbiota can profoundly influence many aspects of host physiology, including regulating metabolism 55, activating the immune system 56, and promoting cancer 57. A previous study confirmed that inducing the expansion of genotoxic abilities and altered microbial composition can promote the development of intestinal tumors 58. The transition from normal epithelium to laryngeal carcinoma consists of extensive, comprehensive, and multiple stages. There are numerous complex linkages between microbiota and the initiation and development of cancer, and microbiota may influence cancer progression in complex ways. Bacteriacan cooperatively form a mixed community that is considered an integrated organization of populations that coexist and interplay within a given niche. Each component plays an effective role in maintaining the ecological balance. Various bacterial combinations may contribute to disease, indicating that collaborative activities of different microbial communities may influence the outcome of the disease 59. Despite the limited number of samples in our study, our results suggest that imbalances in bacterial communities caused by increases or decreases in the abundance of some OTUs may be one of the causes of GLSCC. However, this hypothesis needs to be confirmed by further research.

In summary, we compared bacterial communities in the vocal cord mucous membrane between GLSCC patients and control subjects and identified that the phylum Fusobacteria, the class Fusobacteriia, the order Fusobacteriales, the family Fusobacteriaceae, and the genera *Fusobacterium*, *Alloprevotella*, and *Prevotella*may play a major role in the initiation and progression of GLSCC. Although these conclusions need to be confirmed, our results provide a new research direction for the prevention, diagnosis, and treatment of GLSCC.

## Supplementary Material

Supplementary figures and table.

## Figures and Tables

**Table 1:** Clinical sample data.

Parameters	GLSCC subjects	Control subjects
**Gender**		
Male	18	15
Female	1	6
**Age**		
≤60	6	10
>60	13	11
**Alcohol**		
Yes	16	7
No	3	14
**Smoking**		
Yes	17	10
No	2	11
**T classification**		
T1 and T2	10	**-**
T3 and T4	9	**-**
**Lymph node metastasis**		
Yes	3	**-**
No	16	**-**
**Tumor sizes**		
≤2 cm^3^	8	**-**
>2 cm^3^	11	**-**

**Table 2:** Sequence data with richness, the coverage percentage, and diversity estimation of various bacterial taxa among three groups of vocal cord mucosa.

	Chao1 (median)	Good's coverage (median)	Shannon (median)	Simpson (median)
Control	947.47	0.96	7.50	0.99
NAT	2056.23	0.92	8.19	0.99
Tumor	1016.57	0.96	7.33	0.98
