# Supplementary material for: Alterations of bacterial communities of vocal cord mucous membrane increases the risk for glottic laryngeal squamous cell carcinoma
Source: J Cancer. 2021 May 13;12(13):4049–63. doi: 10.7150/jca.54221 (PMC8176248; doi:10.7150/jca.54221)
Supplement: Supplementary file 1 — Supplementary figures and table. [file jcav12p4049s1.pdf]

Figure S1

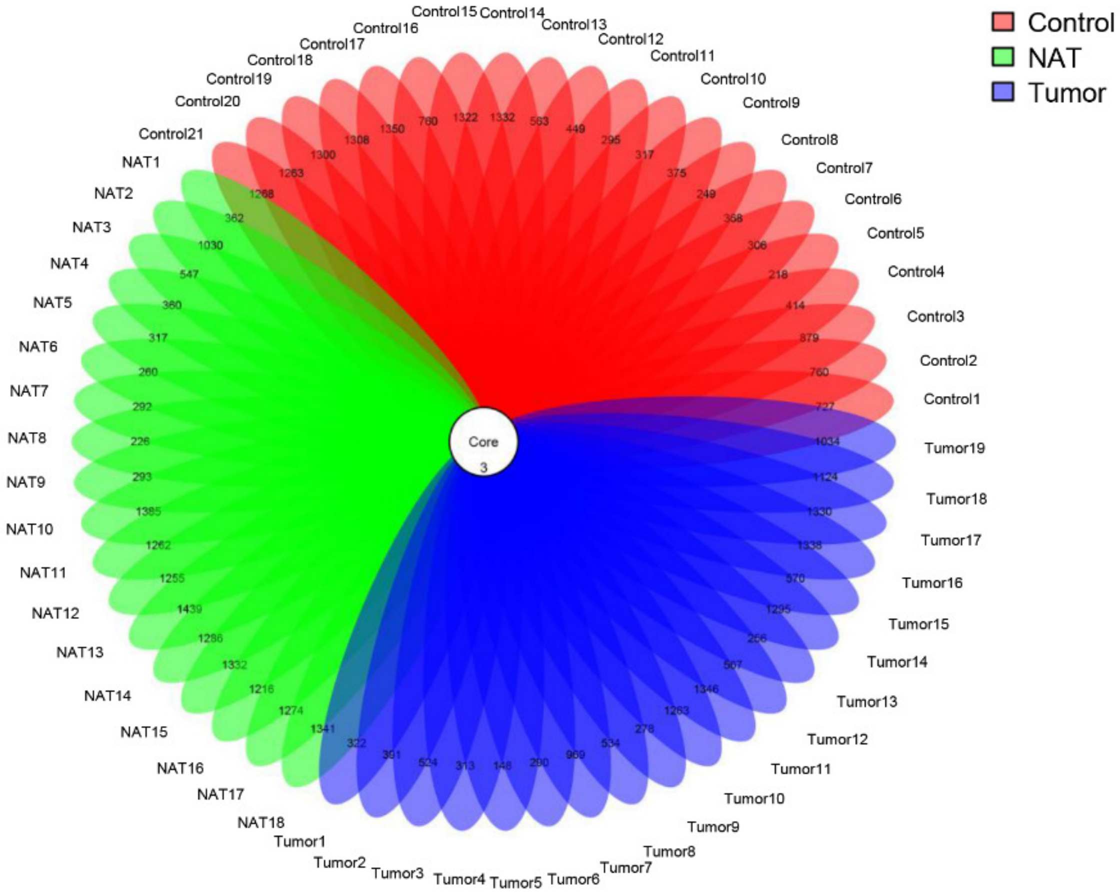

Figure S1: The number in the core represents the OTUs that are common to all samples (Core OTUs), and the number on the petals indicates the total OTUs of every sample minus the number of OTUs that are common to all samples.

Figure S2

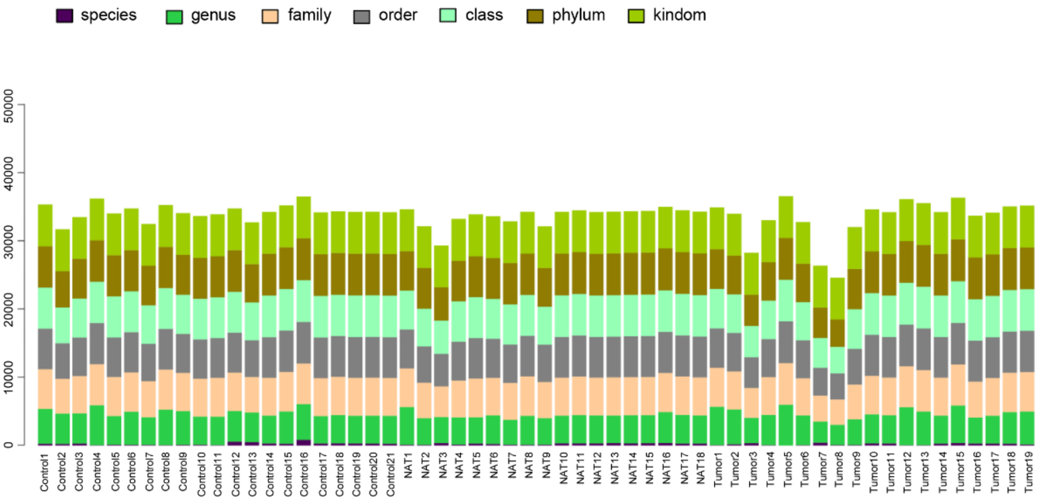

Figure S2: The total number of tags in the OTU of each level of classification to the kingdom, phylum, class, order, family, genus, and specie were summarized to obtain the OTU level barplot of annotation proportion of each sample. The horizontal axis is the sample name, that is, a column represents a sample, and the vertical axis is the total tags statistics in OTUs annotated at different classification levels.

Supplemental Table 1. Bacteria identified (top 15) in the normal adjacent tissue (NAT) samples

Phylum	Class	Order	Family	Genus
Bacteroidetes	Bacteroidia	Bacteroidales	Mycoplasmataceae	Mycoplasma
Proteobacteria	Mollicutes	Mycoplasmatales	Prevotellaceae	Streptococcus
Firmicutes	Clostridia	Clostridiales	Streptococcaceae	Bacteroides
Tenericutes	Bacilli	Lactobacillales	Bacteroidaceae	Fusobacterium
Actinobacteria	Gammaproteobacteria	Fusobacteriales	Fusobacteriaceae	uncultured_bacterium
Fusobacteria	Betaproteobacteria	Burkholderiales	Lachnospiraceae	uncultured
Gemmatimonadetes	Alphaproteobacteria	Pasteurellales	Bacteroidales_S24_7_group	Prevotella_9
Acidobacteria	Fusobacteriia	Enterobacteriales	Ruminococcaceae	Alloprevotella
Cyanobacteria	Actinobacteria	Sphingomonadales	Pasteurellaceae	Haemophilus
Spirochaetae	Deltaproteobacteria	Neisseriales	Enterobacteriaceae	Faecalibacterium
Nitrospirae	Erysipelotrichia	Pseudomonadales	Porphyromonadaceae	Escherichia_Shigella
Chloroflexi	Gemmatimonadetes	Rhodospirillales	Comamonadaceae	Enterococcus
Chlorobi	Sphingobacteriia	Rhizobiales	Neisseriaceae	Porphyromonas
Candidate_division_SR1	Epsilonproteobacteria	Micrococcales	Peptostreptococcaceae	Prevotella
Fibrobacteres	Acidimicrobiia	Xanthomonadales	Erysipelotrichaceae	Neisseria
